# Impact of smoke-free policies in hospitality venues and the home environment on smoking behaviour and exposure to second-hand smoke: results of two systematic reviews

**DOI:** 10.1093/eurpub/ckac129.755

**Published:** 2022-10-25

**Authors:** YY da Costa Senior, AD Rozema, AE Kunst, MAG Kuipers

**Affiliations:** 1 Department of Public and Occupational Health, Amsterdam UMC, University of Amsterdam, Amsterdam, Netherlands; 2 Tranzo, Tilburg University, Tilburg, Netherlands

## Abstract

**Background:**

Smoke-free policies (SFPs) have proven to be effective in protecting people from exposure to second-hand smoke (SHS) and lowering smoking rates. Our aims were to assess the impact of SFPs in hospitality venues (e.g. bars) on smoking behaviour of young people and to assess the impact of SFPs in the home environment on smoking behaviour and exposure to SHS.

**Methods:**

Two reviews were conducted. The first was conducted in June 2020. We searched PubMed, Embase, and Scopus for studies that assessed the association between any form of SFPs in hospitality venues and a measure of smoking behaviour among young people (aged 10-24 years). The second review will be conducted in June 2022. Searches will be conducted in PubMed, Embase, Web of Science, PsycINFO and CENTRAL. We will search for studies that assess the association between any form of SFPs in the home environment (e.g. multi-unit housing) and a measure of smoking behaviour (e.g. initiation) or SHS exposure.

**Results:**

Nine studies (publication years 2005-2016) were included in the first review, of which the majority used a quasi-experimental design. Four studies evaluated SFPs in hospitality venues specifically. Two studies reported that strict, but not weaker, SFPs decrease progression to established smoking. Two other studies provided mixed results. Five studies also included other workplaces, of which three studies found significant decreases in current smoking, smoking frequency, and/or smoking quantity. The results of the second review will be presented in detail during the workshop, however an exploration suggests that SFPs in the home environment may prevent smoking and SHS exposure.

**Conclusions:**

Most studies of the first review found that SFPs in hospitality venues are associated with a decrease in smoking behaviour among young people. Their results indicate the need for strict smoke-free legislation without exemptions. The conclusions of the second review will be presented during the workshop.

